# MRI of Knee Joint Lesions: An Observational Study of Traumatic, Degenerative, Cystic, and Neoplastic Pathologies

**DOI:** 10.7759/cureus.106407

**Published:** 2026-04-03

**Authors:** Tanushri Ghosh, Subhankar Choudhury, Anand Kumar, Pramod Kumar

**Affiliations:** 1 Department of Radiodiagnosis, Hi-Tech Medical College and Hospital, Rourkela, IND; 2 Department of Pharmacology, Hi-Tech Medical College and Hospital, Rourkela, IND; 3 Department of Forensic Medicine and Toxicology, Hi-Tech Medical College and Hospital, Rourkela, IND; 4 Department of Biochemistry, Hi-Tech Medical College and Hospital, Rourkela, IND

**Keywords:** anterior cruciate ligament injury, baker’s cyst, knee joint pathology, magnetic resonance imaging, meniscus tear, synovial sarcoma

## Abstract

Background

Knee joint disorders are a major cause of musculoskeletal morbidity and functional disability worldwide, involving a broad spectrum of traumatic, degenerative, cystic, and neoplastic pathologies. Accurate evaluation of these conditions is essential for appropriate clinical assessment. MRI provides detailed visualization of intra-articular and periarticular structures due to its superior soft tissue contrast and multiplanar capability. However, there remains a need for region-specific observational data describing the spectrum of MRI findings in routine clinical practice.

Materials and methods

This retrospective observational study was conducted in the Department of Radiodiagnosis at Hi-Tech Medical College and Hospital, Rourkela, India, following institutional ethics approval. A total of 236 patients who underwent MRI of the knee between August 2024 and July 2025 were included. Patients referred with clinical suspicion of knee joint pathology were evaluated. MRI findings were analyzed to determine the distribution of knee joint pathologies and their association with demographic variables.

Results

Of the 236 patients, 149 (63.13%) were males, and 87 (36.87%) were females. Ligamentous and meniscal injuries were the most common findings, observed in 134 patients (56.78%), with a predominance among younger male patients. Anterior cruciate ligament tears were the most frequent ligamentous injury, followed by medial meniscus tears. Osteoarthritis was identified in 47 cases (19.92%) and was more frequently observed in older female patients. Baker’s cysts were observed in 25 cases (10.59%), fractures in 20 cases (8.47%), and malignant neoplasms in 10 cases (4.24%). Among fractures, patellar fractures were the most common pattern. Among malignant lesions, synovial sarcoma was the most frequently identified tumor.

Conclusions

Knee joint pathologies demonstrated a distinct distribution pattern, with ligamentous and meniscal injuries predominating in younger males and degenerative changes more frequent in older females. Within the framework of this observational study, MRI facilitated detailed characterization of a wide spectrum of knee abnormalities; however, in the absence of comparative or outcome-based data, conclusions regarding diagnostic accuracy or clinical impact cannot be established. These findings provide context-specific insights into the epidemiological and imaging profile of knee joint pathologies and may serve as a foundation for future prospective and comparative studies.

## Introduction

Knee joint pathology represents a significant cause of musculoskeletal morbidity and functional disability worldwide, affecting individuals across a broad demographic spectrum, including young athletes, middle-aged individuals, and the elderly population. Given its complex anatomical structure and essential role in weight-bearing, stability, and locomotion, the knee joint is particularly vulnerable to a wide range of pathological conditions. These disorders commonly present with pain, swelling, stiffness, and functional limitation, thereby contributing substantially to reduced quality of life and increased healthcare burden [1].

Knee joint pathologies can be broadly classified into traumatic, degenerative, cystic, and neoplastic lesions, each characterized by distinct etiological factors, clinical presentations, and imaging features [2]. Traumatic lesions frequently involve ligamentous structures, menisci, and osseous components and are typically associated with twisting injuries, direct trauma, or high-energy mechanisms. Degenerative conditions, particularly osteoarthritis, represent the most prevalent category and are associated with aging, obesity, hormonal influences, and chronic mechanical stress [3,4]. Cystic lesions of the knee, including Baker’s cysts, parameniscal cysts, and ganglion cysts, are often secondary to underlying intra-articular pathology and may contribute to pain, swelling, and restricted movement [5]. Neoplastic lesions, although relatively less common, are clinically significant due to their potential for local invasion and distant spread and include benign tumors such as osteochondroma as well as malignant entities such as osteosarcoma and synovial sarcoma, which require accurate imaging characterization for appropriate management [6].

Accurate diagnosis of knee joint pathology is essential for guiding clinical management and optimizing patient outcomes. However, clinical examination alone may be limited, particularly in acute settings due to pain, swelling, and restricted mobility [7]. Conventional imaging modalities such as radiography and ultrasonography, although widely used, have limited sensitivity for evaluating intra-articular structures. CT provides excellent visualization of osseous anatomy but lacks adequate soft tissue contrast, while arthroscopy, although considered a reference standard for intra-articular evaluation, is invasive and associated with procedural risks [8-11].

In this context, MRI has emerged as a key imaging modality for the comprehensive evaluation of knee joint pathologies. MRI provides superior soft tissue contrast and multiplanar imaging capability, enabling detailed assessment of ligaments, menisci, cartilage, bone marrow, and periarticular soft tissues. In contemporary clinical practice, MRI also plays an important role in treatment planning, surgical decision-making, and monitoring of disease progression, thereby extending its utility beyond diagnosis alone [12].

Despite its established clinical utility, there remains a need for systematic observational studies that evaluate the spectrum of MRI findings in knee joint pathologies in routine clinical practice, particularly in diverse and region-specific populations. Such studies can provide valuable insights into the distribution of lesions, demographic associations, and imaging characteristics within real-world clinical settings.

In this context, the present study was designed to determine the frequency and distribution of knee joint pathologies identified on MRI in a tertiary care setting. In addition, the study aimed to characterize MRI grading patterns of ligamentous and meniscal injuries, assess associations between demographic variables and the type and severity of knee joint pathologies, evaluate the spectrum of traumatic, degenerative, cystic, and neoplastic lesions, and correlate imaging findings with clinical presentation within the scope of an observational study.

## Materials and methods

This retrospective observational study was conducted in the Department of Radiodiagnosis at Hi-Tech Medical College and Hospital, Rourkela, India, following approval from the Institutional Ethics Committee. All patient data were anonymized prior to analysis to ensure confidentiality. The study was carried out over a one-year period from August 1, 2024 to July 31, 2025 and included patients referred from the outpatient department with clinical suspicion of knee joint pathology. As this was a single-center study, the findings may reflect local patient demographics and referral patterns, which may limit generalizability to broader populations.

The sample size was initially estimated using Cochran’s formula:

$n = \frac{Z^2 \, p \, q}{d^2},$

where Z = 1.96 for a 95% confidence level, p represents the estimated prevalence of knee joint pathologies based on prior literature [13], q = 1 − p, and d is the 5% margin of error.

Based on these parameters, the minimum required sample size was calculated prior to study initiation. A consecutive sampling technique was employed, and all eligible patients undergoing MRI during the study period were included. A total of 263 patients underwent MRI of the knee, of whom 27 were excluded due to incomplete data, resulting in a final sample of 236 patients.

The study included patients aged 16-80 years of either sex who were referred for MRI of the knee with clinical suspicion of knee joint pathology. The lower age limit of 16 years was selected to include late adolescents with near skeletal maturity, thereby ensuring comparability with adult knee pathology patterns. Patients with a history of prior knee surgery, incomplete imaging or clinical data, and inpatient cases were excluded. The exclusion of inpatient cases was intended to maintain a relatively uniform outpatient cohort; however, this may have resulted in underrepresentation of high-energy traumatic injuries and is acknowledged as a potential source of selection bias. Patients with inflammatory arthropathies were not specifically excluded, which may have influenced certain imaging findings.

MRI examinations were performed using a 1.5 Tesla scanner (Signa; General Electric Medical Systems, Inc., Milwaukee, WI, USA) with the patient in the supine position using a dedicated knee coil. The imaging protocol included T1-weighted sequences in sagittal and coronal planes, proton density fat-suppressed (PD-FS) sequences in sagittal and coronal planes, T2-weighted sequences in axial, sagittal, and coronal planes, and short tau inversion recovery sequences. Typical imaging parameters included a slice thickness of approximately 3-4 mm, a field of view of 14-16 cm, and a matrix size of approximately 256 × 256, consistent with standard clinical protocols. The use of a 1.5 Tesla system may limit sensitivity for detecting early cartilage changes compared with higher-field systems. Advanced quantitative cartilage imaging techniques such as T2 mapping, T2* mapping, or delayed gadolinium-enhanced MRI of cartilage were not available and were therefore not included. Contrast-enhanced sequences were also not performed, which may limit detailed evaluation of synovial pathology and certain neoplastic lesions.

MRI images were systematically evaluated for abnormalities involving ligaments, menisci, bones, cartilage, and surrounding soft tissues. Ligamentous injuries involving the anterior cruciate ligament (ACL), posterior cruciate ligament (PCL), and medial collateral ligament (MCL) were graded based on standard MRI criteria as Grade I (edema with intact fibers), Grade II (partial fiber disruption), and Grade III (complete tear) [12]. Meniscal tears were graded according to the Stoller classification system [14], and osteoarthritis was assessed using the Kellgren-Lawrence (KL) grading system [15]. Fractures were classified using established systems, including the Salter-Harris and Schatzker classifications [16]. Image interpretation was performed by a qualified radiologist, and due to the retrospective nature of the study, formal interobserver variability assessment was not conducted, which is acknowledged as a limitation. Cartilage lesions and bone marrow edema, although detectable on the sequences used, were not systematically graded using standardized scoring systems, which is also recognized as a limitation.

Data were collected using a structured pro forma, including demographic variables such as age, sex, BMI, and socioeconomic status, along with clinical presentation and MRI findings. Socioeconomic status was categorized according to the Updated B.G. Prasad Socioeconomic Classification for 2025 [17]. BMI was calculated by dividing weight in kilograms by height in meters squared (kg/m²) and categorized according to the Asian-Indian-specific cutoffs as established by the 2009 Indian Consensus Statement (Table 1) [18].

**Table 1:** BMI categories Asian-Indian specific cutoffs as established by the 2009 Indian Consensus Statement [17]

BMI (kg/m²)	Category
<18.5	Underweight
18.5-22.9	Normal
23-24.9	Overweight
25-29.9	Obese Class I
≥30	Obese Class II

Statistical analysis was performed using Microsoft Excel 2021 (Microsoft Corporation, Redmond, WA, USA). Categorical variables were expressed as frequencies and percentages, and associations between variables were assessed using the chi-square test. When expected cell counts were less than five, Fisher’s exact test or Fisher-Freeman-Halton exact test was applied. A p-value of less than 0.05 was considered statistically significant. The analysis was primarily descriptive and exploratory. Multivariate analysis and adjustment for multiple comparisons were not performed and are acknowledged as limitations. The study was conducted and reported in accordance with the STrengthening the Reporting of OBservational studies in Epidemiology (STROBE) guidelines for observational studies to ensure methodological transparency and completeness.

## Results

In the present study, the demographic characteristics of the study population, including age group, sex, marital status, religion, employment status, socioeconomic status, and BMI, were obtained from the medical records of the patients. A total of 236 patients were included in the analysis, of whom 149 (63.13%) were males, and 87 (36.87%) were females. The age of patients ranged from 16 to 80 years. Among males, the highest proportion belonged to the 21-30 years age group (41 cases, 17.37%), followed by the 31-40 years age group (37 cases, 15.68%) and the 61-70 years age group (29 cases, 12.29%). Among females, the 41-50 years age group constituted the largest proportion (29 cases, 12.29%), followed by the 51-60 years age group (15 cases, 6.36%) (Table 2).

**Table 2:** Demographic characteristics of the study population Socioeconomic status classified according to the Updated B.G. Prasad Socioeconomic Classification for 2025 [16].

Parameter	Category	Male, n (%)	Female, n (%)
Age group (years)	16-20	4 (1.69)	3 (1.27)
	21-30	41 (17.37)	16 (6.78)
	31-40	37 (15.68)	13 (5.51)
	41-50	20 (8.47)	29 (12.29)
	51-60	11 (4.66)	15 (6.36)
	61-70	29 (12.29)	8 (3.39)
	71-80	7 (2.97)	3 (1.27)
	Total	149 (63.13)	87 (36.87)
BMI	Underweight	6 (2.54)	5 (2.12)
	Normal	43 (18.22)	16 (6.78)
	Overweight	36 (15.25)	27 (11.44)
	Obese Class I	33 (13.98)	21 (8.90)
	Obese Class II	31 (13.14)	18 (7.63)
	Total	149 (63.13)	87 (36.87)
Marital status	Single	42 (17.80)	11 (4.66)
	Married	71 (30.08)	51 (21.62)
	Separated	16 (6.78)	12 (5.09)
	Divorced	11 (4.66)	9 (3.81)
	Widowed	9 (3.81)	4 (1.69)
	Total	149 (63.13)	87 (36.87)
Religion	Hindu	83 (35.17)	53 (22.46)
	Muslim	34 (14.41)	19 (8.06)
	Christian	21 (8.89)	8 (3.39)
	Sikh	6 (2.54)	4 (1.69)
	Jain	5 (2.12)	3 (1.27)
	Total	149 (63.13)	87 (36.87)
Employment status	Not employed	23 (9.74)	47 (19.92)
	Part time	72 (30.51)	25 (10.59)
	Full time	54 (22.88)	15 (6.36)
	Total	149 (63.13)	87 (36.87)
Socioeconomic status	Class I (upper)	17 (7.20)	9 (3.81)
	Class II (upper middle)	26 (11.02)	13 (5.51)
	Class III (middle)	44 (18.64)	29 (12.29)
	Class IV (lower middle)	39 (16.52)	17 (7.20)
	Class V (lower)	23 (9.75)	19 (8.06)
	Total	149 (63.13)	87 (36.87)

Regarding BMI, a substantial proportion of patients were either overweight or obese. Specifically, 63 patients (26.69%) were overweight, 54 (22.88%) belonged to obese Class I, and 49 (20.76%) belonged to obese Class II. Only 59 patients (25.00%) had normal BMI, while 11 patients (4.66%) were underweight. Notably, 70.33% of the study population had a BMI above the normal range (Table 2).

The majority of patients were married (122 cases, 51.69%), followed by single individuals (53 cases, 22.46%). Smaller proportions were separated (28 cases, 11.87%), divorced (20 cases, 8.47%), or widowed (13 cases, 5.51%). In terms of religion, most patients belonged to the Hindu religion (136 cases, 57.63%), followed by Muslims (53 cases, 22.46%), Christians (29 cases, 12.29%), Sikhs (10 cases, 4.23%), and Jains (eight cases, 3.39%) (Table 2).

Regarding employment status, the largest proportion of patients were part-time employed (97 cases, 41.10%), followed by full-time employed (69 cases, 29.24%), while 70 patients (29.66%) were unemployed. The socioeconomic distribution showed that the majority belonged to the middle class (Class III: 73 cases, 30.93%), followed by lower middle class (Class IV: 56 cases, 23.72%), upper middle class (Class II: 39 cases, 16.53%), lower class (Class V: 42 cases, 17.80%), and upper class (Class I: 26 cases, 11.02%) (Table 2).

**Figure 1:**
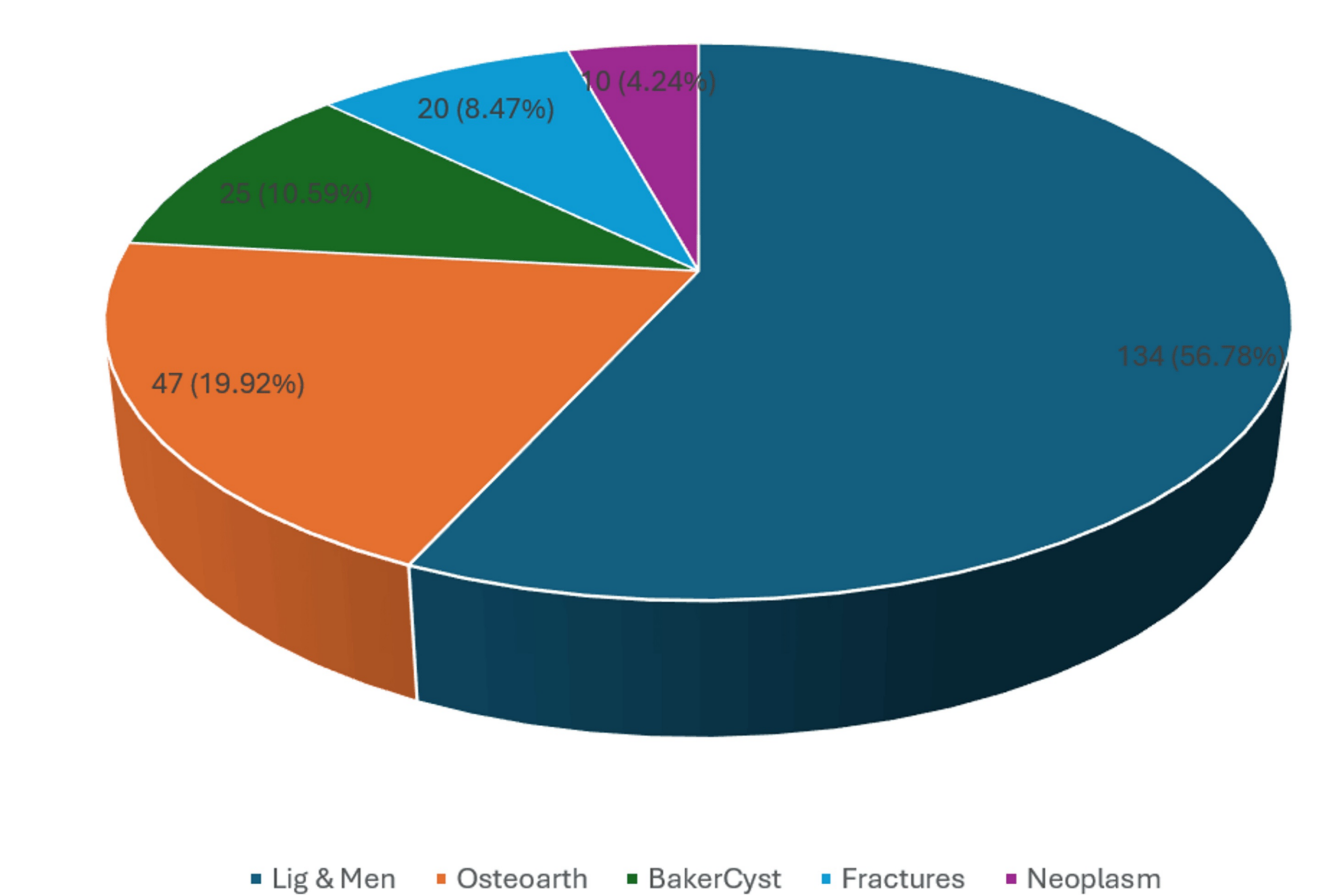
Distribution of knee joint lesions according to their pathology

Among the 236 cases, the most common pathology detected on MRI was ligament and meniscal injuries (134 cases, 56.78%), followed by degenerative osteoarthritis (47 cases, 19.92%), Baker’s cysts (25 cases, 10.59%), fractures around the knee joint (20 cases, 8.47%), and malignant neoplasms (10 cases, 4.24%) (Figure 1).

A chi-square test revealed a statistically significant association between sex and type of knee joint lesion (χ² = 13.47, df = 4, p = 0.009). Ligament and meniscal injuries were significantly more common among males (63.76% of male cases) compared to females (44.82% of female cases), whereas osteoarthritis was relatively more frequent among females (33.33% of female cases) compared to males (12.08% of male cases) (Table 3). 

**Table 3:** Association between sex and knee joint lesions

Lesion type	Male	Female	Total
Ligament and meniscal injuries	95	39	134
Osteoarthritis	18	29	47
Baker’s cyst	16	9	25
Fractures	15	5	20
Malignant neoplasms	8	2	10
Total	149	87	236

**Figure 2:**
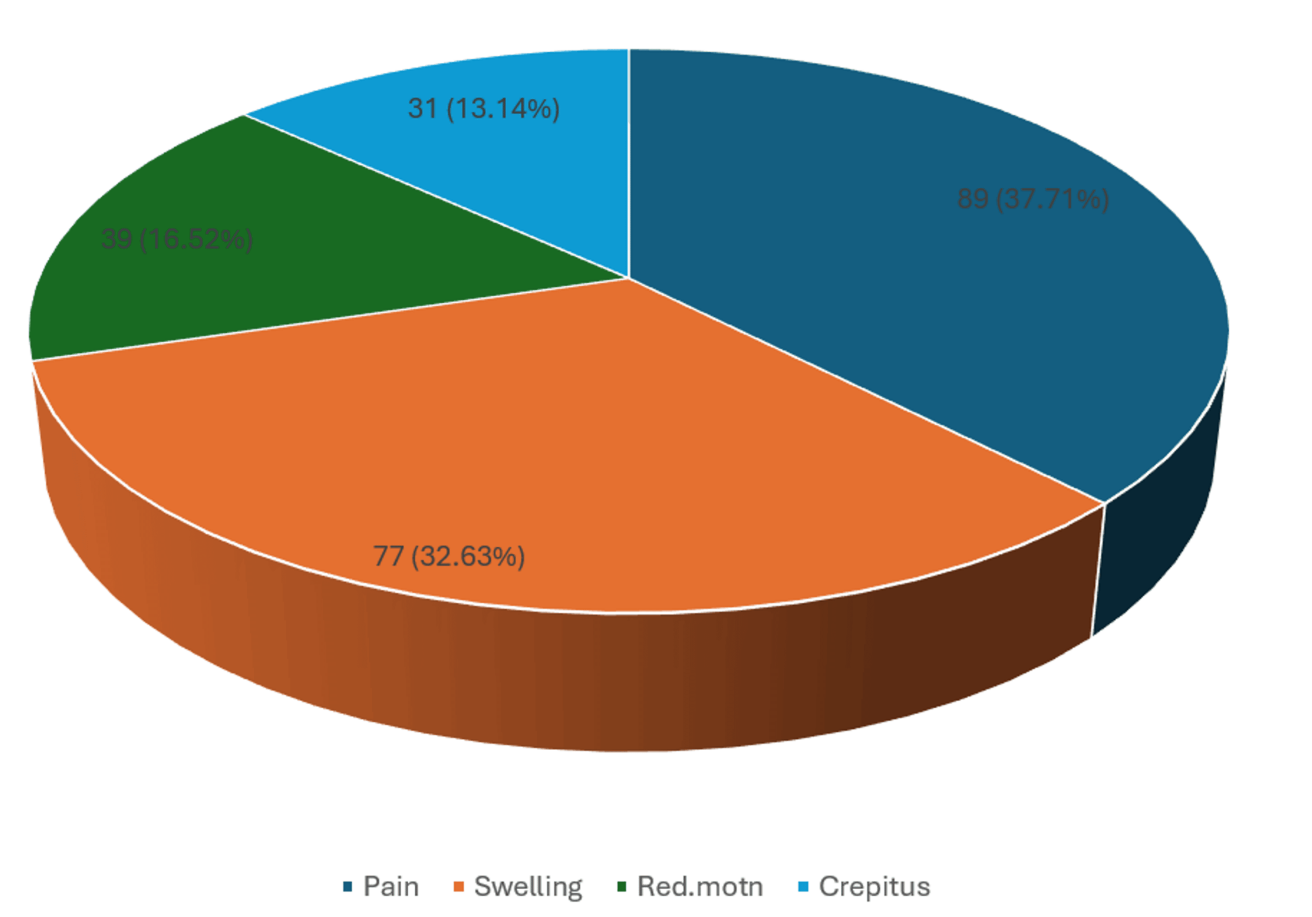
Most common presenting symptoms of knee joint lesions

Pain was the most frequent presenting symptom, observed in 89 patients (37.71%), followed by swelling in 77 patients (32.63%), reduced range of motion in 39 patients (16.52%), and crepitus in 31 patients (13.14%) (Figure 2).

**Figure 3:**
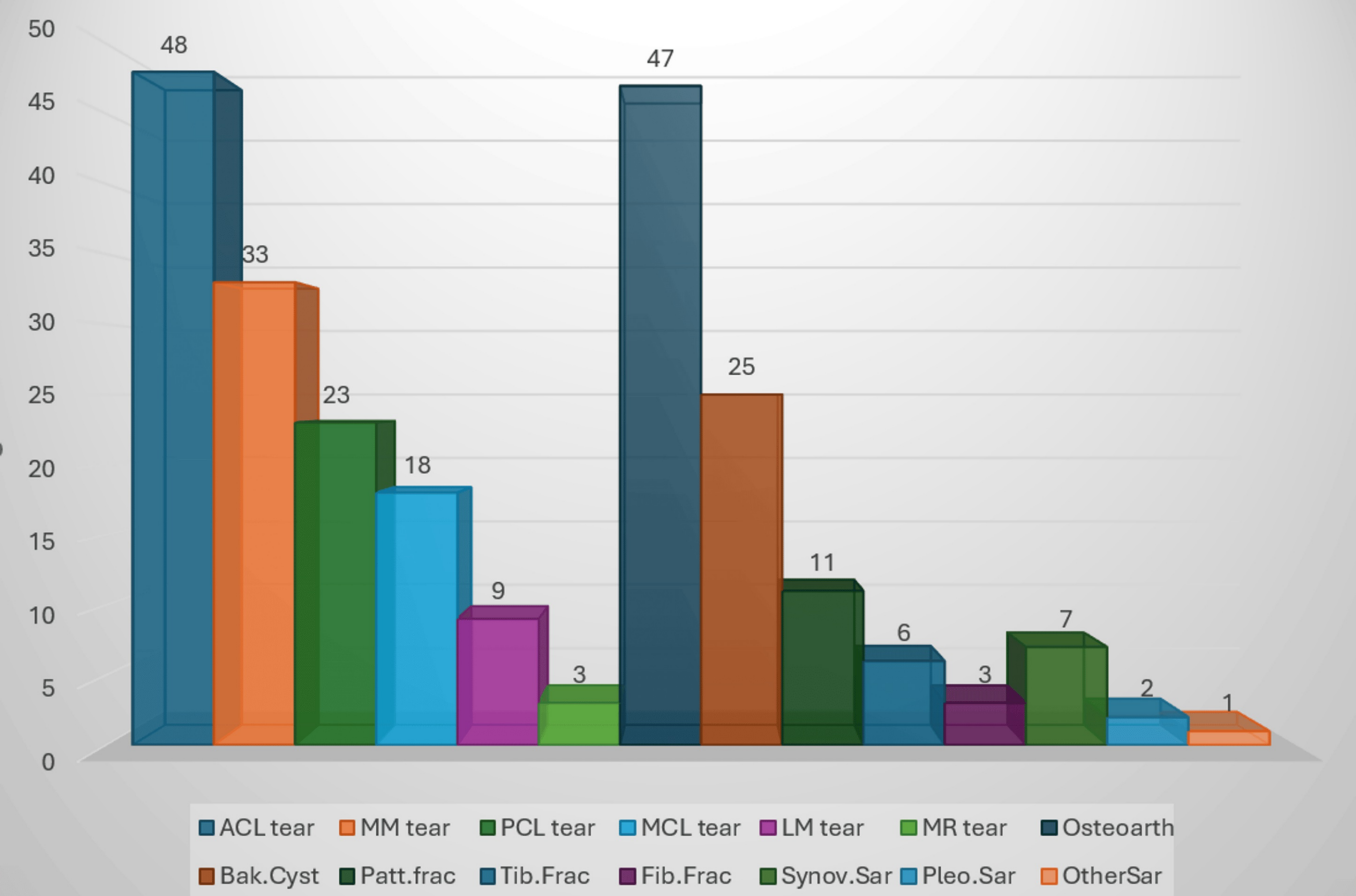
Distribution of knee joint lesions according to their individual incidences

Among the 134 ligament and meniscal injuries, ACL tears were the most common (48 cases, 20.34%), followed by medial meniscus (MM) tears (33 cases, 13.98%), PCL tears (23 cases, 9.75%), MCL injuries (18 cases, 7.63%), lateral meniscus (LM) tears (nine cases, 3.81%), and medial retinaculum tears (three cases, 1.27%). Next was osteoarthritis (47 cases, 19.92%), followed by Baker’s cyst (10.59%). Among traumatic fractures, patellar fractures were the highest (11 cases, 4.66%). It was followed by tibial fractures (six cases, 2.54%) and fibular fractures (three cases, 1.27%). In malignant neoplasms, the highest incidence was of synovial sarcomas (seven cases, 2.97%), followed by pleomorphic sarcomas (two cases, 0.85%) and one other soft tissue sarcoma (one case, 0.42%) (Figure 3).

The MRI grading pattern of ligament and meniscal injuries is detailed in Table 4. Among ACL tears, Grade II tears were most common (18 cases, 7.63%), followed by Grade III (16 cases, 6.78%) and Grade I (14 cases, 5.93%). For PCL tears, Grade III was most frequent (12 cases, 5.08%), followed by Grade II (eight cases, 3.39%). Similarly, for MM tears, Grade III predominated (17 cases, 7.20%), followed by Grade II (11 cases, 4.66%). In MCL injuries, Grade I tears were most common (16 cases, 6.78%), with only two cases of Grade II and no Grade III tears. For LM tears, Grade III was most frequent (four cases, 1.69%). All medial retinaculum tears were Grade II (three cases). Due to the small sample size of medial retinaculum injuries, this category was excluded from the chi-square analysis to avoid violation of expected frequency assumptions. A chi-square test demonstrated a significant association between ligament type and MRI grading pattern (χ² = 13.56, df = 8, p = 0.009), indicating that different ligament structures exhibit distinct patterns of injury severity (Table 4).

**Table 4:** MRI grading of knee joint ligament injuries ACL, anterior cruciate ligament; LM, lateral meniscus; MCL, medial collateral ligament; MM, medial meniscus; PCL, posterior cruciate ligament

Lesions	Sex	Grade I	Grade II	Grade III	Total
ACL tear	Male	11	12	12	35
	Female	3	6	4	13
	Total	14	18	16	48
MM tear	Male	3	7	13	23
	Female	2	4	4	10
	Total	5	11	17	33
PCL tear	Male	2	7	9	18
	Female	1	1	3	5
	Total	3	8	12	23
MCL tear	Male	11	1	0	12
	Female	5	1	0	6
	Total	16	2	0	18
LM tear	Male	2	1	3	6
	Female	1	1	1	3
	Total	3	2	4	9
Medial retinaculum tear	Male	0	1	0	1
	Female	0	2	0	2
	Total	0	3	0	3

Among the 47 cases of osteoarthritis, Grade II osteoarthritis was the most common (32 cases, 13.56%), followed by Grade I (eight cases, 3.39%), Grade III (five cases, 2.12%), and Grade IV (two cases, 0.85%) according to the KL grading system. Females showed a higher proportion of osteoarthritis cases (29 cases, 33.33% of all female patients) compared to males (18 cases, 12.08% of all male patients). However, no statistically significant association was observed between sex and severity of osteoarthritis as measured by KL grade (Table 5).

**Table 5:** Distribution of osteoarthritis according to KL grade and sex KL grading system [14] KL, Kellgren-Lawrence

KL grade	Male (n)	Female (n)	Total (n)
Grade 1	2	6	8
Grade 2	13	19	32
Grade 3	2	3	5
Grade 4	1	1	2
Total	18	29	47

Statistical analysis also did not demonstrate a significant association between BMI category and severity of osteoarthritis. Nevertheless, a higher proportion of osteoarthritis patients were either overweight or obese (85.11%) compared with those having normal or below-normal BMI (14.89%) among 47 cases (Table 6).

**Table 6:** Distribution of osteoarthritis according to KL grade and BMI category KL grading system [14]. Asian-Indian specific cutoffs as established by the 2009 Indian Consensus Statement [17]. KL, Kellgren-Lawrence

BMI category	Grade 1	Grade 2	Grade 3	Grade 4	Total
Underweight	0	1	0	0	1
Normal	1	3	1	0	5
Overweight	3	8	1	0	12
Obese Class I	2	11	2	1	16
Obese Class II	2	9	1	1	13
Total	8	32	5	2	47

Among the 20 cases of fractures, patellar fractures were the most common (11 cases, 4.66%), followed by fractures of the upper end of the tibia (six cases, 2.54%) and fibular upper end fractures (three cases, 1.27%). Among patellar fractures, transverse fractures were most frequent (six cases), followed by vertical fractures (three cases) and marginal fractures (two cases). Tibial fractures included upper-ended plateau fractures (three cases), epiphyseal fractures (two cases), and a tubercle fracture (one case). Fibular fractures comprised transverse (one case), oblique (one case), and stress fractures (one case) (Table 7).

**Table 7:** Distribution of fractures around the knee joint

Lesions	Subtypes			Total
Patellar fracture	Transverse	Vertical	Marginal	
	6	3	2	11
Tibial fracture	Epiphyseal	Upper end	Tubercle	
	2	3	1	6
Fibular fracture	Transverse	Oblique	Stress	
	1	1	1	3

Among the 10 malignant neoplasms, synovial sarcoma was the most frequent (seven cases), followed by pleomorphic sarcoma (two cases) and soft tissue sarcoma (one case).

Illustrative MRI cases of knee joint lesions

Case 1

**Figure 4:**
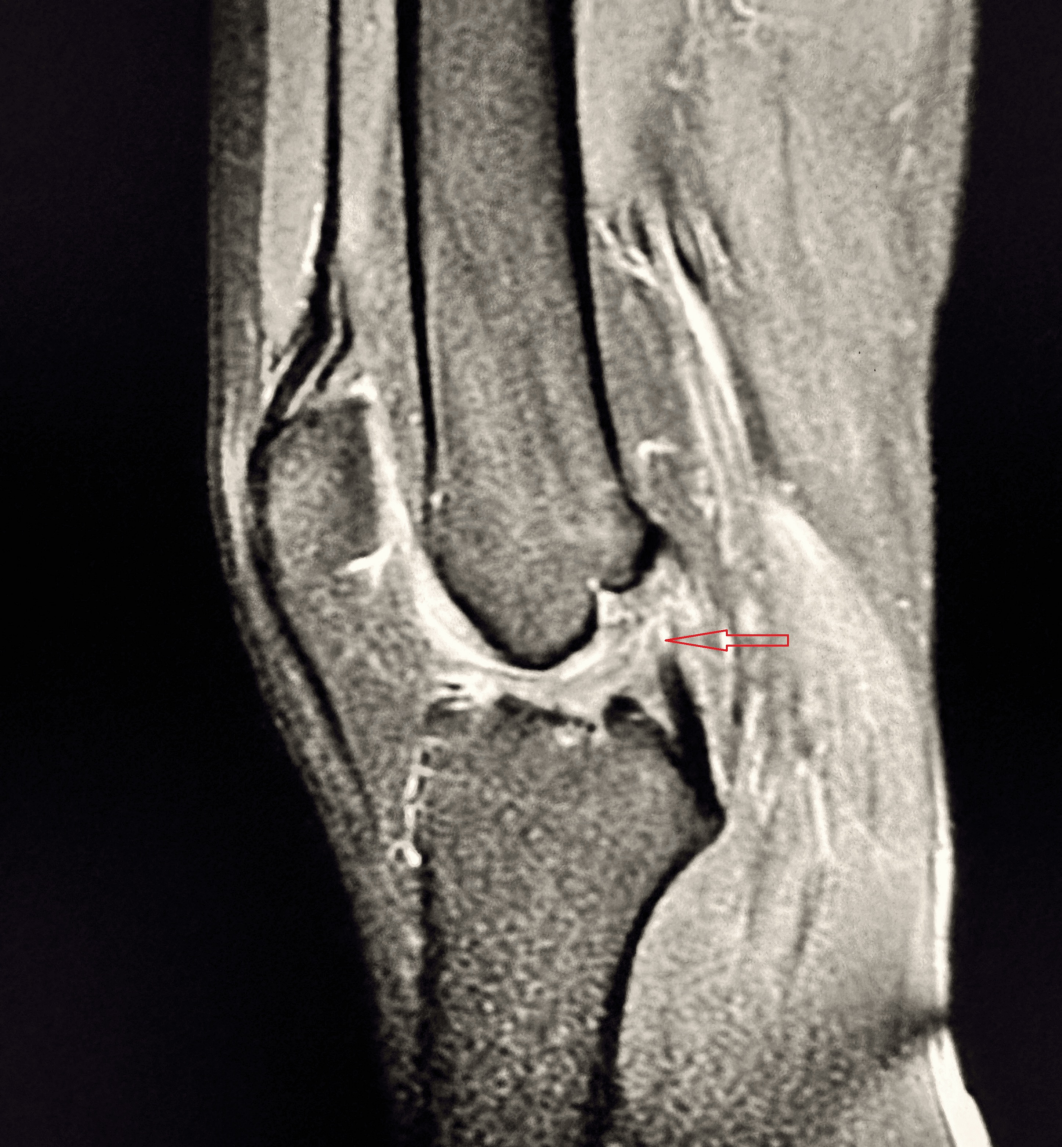
Grade II (partial) tear of the ACL Sagittal PD-FS MRI demonstrates a bulky, hyperintense ACL with loss of normal fibrillar architecture, consistent with a partial Grade II tear (red arrow). Mild joint effusion is present. ACL, anterior cruciate ligament; PD-FS, proton density fat-suppressed

A 19-year-old male presented with pain and instability in the left knee after slipping on rocks while crossing a waterfall during a picnic. Clinical examination revealed positive Lachman test, anterior drawer test, and pivot shift test, suggestive of ACL injury. MRI confirmed a Grade II ACL tear (Figure 4).

Case 2

**Figure 5:**
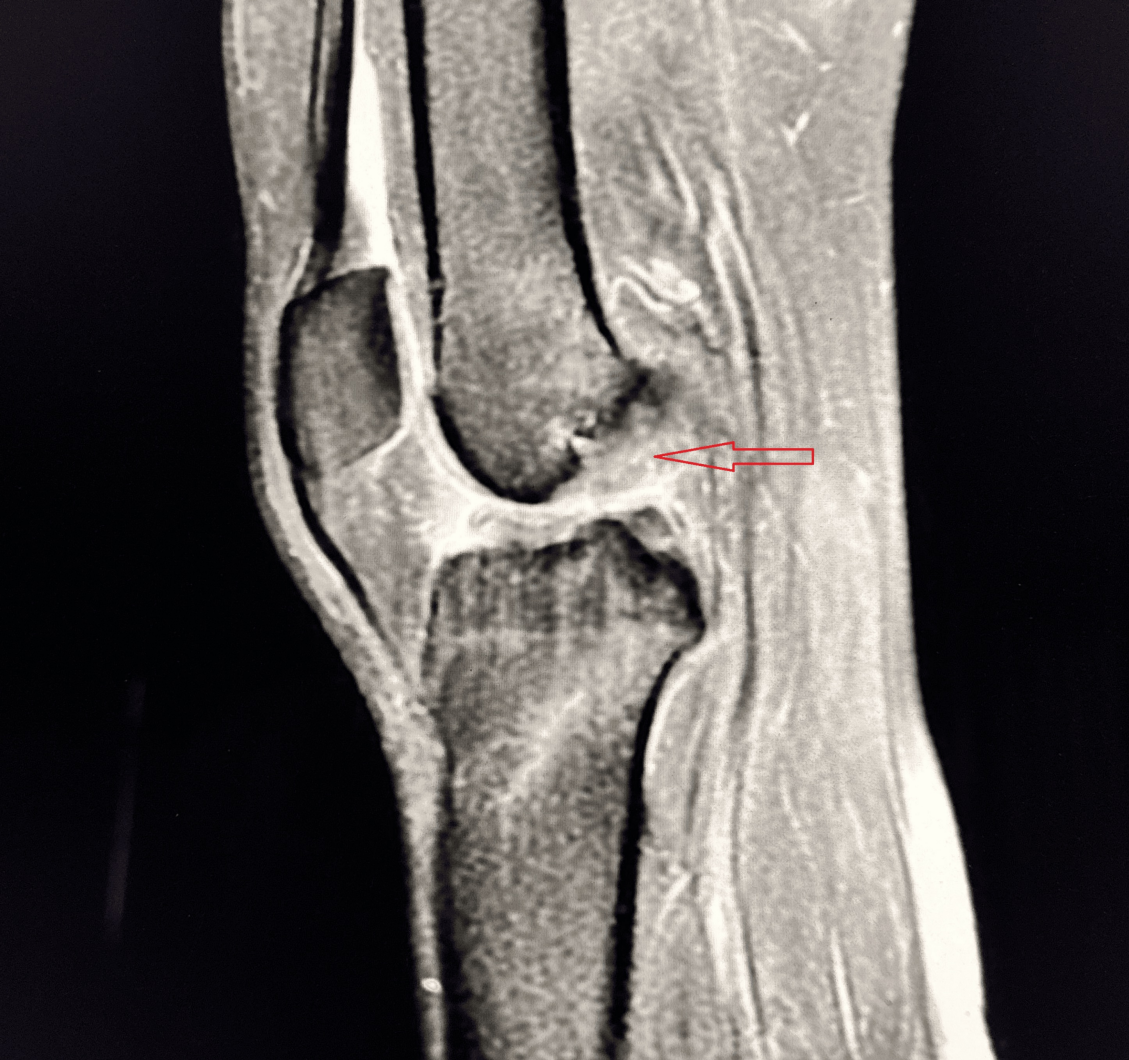
Grade III (complete) tear of the ACL Sagittal PD-FS MRI shows complete discontinuity of ACL fibers with marked hyperintensity, consistent with a complete Grade III ACL rupture (red arrow). Moderate knee joint effusion is also present. ACL, anterior cruciate ligament; PD-FS, proton density fat-suppressed

A 25-year-old male presented following a motor vehicle accident with severe pain, swelling, and restricted movement of the left knee joint. Physical examination was limited due to acute pain. MRI demonstrated a complete Grade III ACL tear (Figure 5).

Case 3

**Figure 6:**
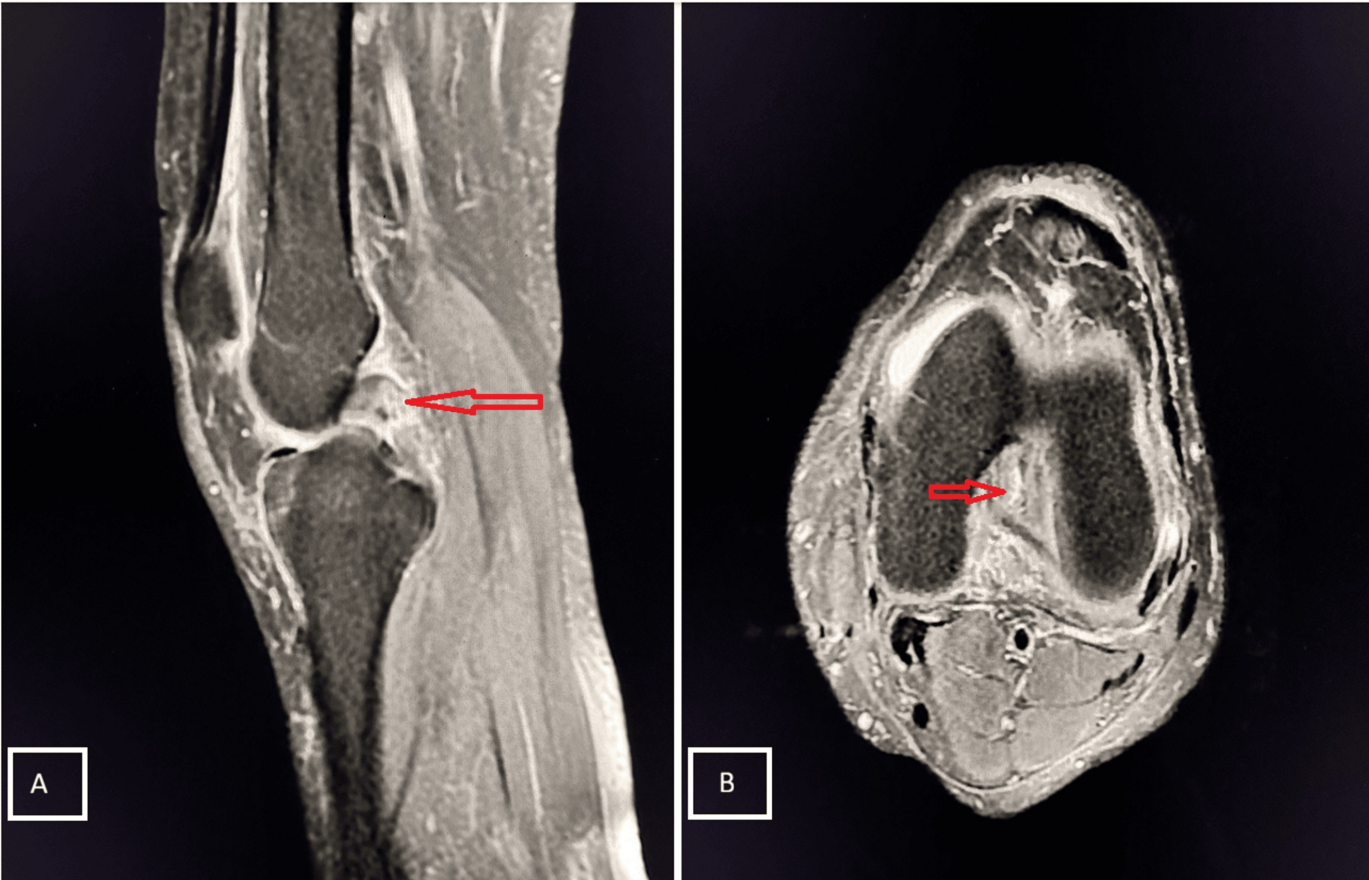
Grade III tear of the PCL Sagittal PD-FS (A) and axial (B) MRI demonstrates a hyperintense, thickened PCL with complete disruption at the mid-portion, consistent with a Grade III PCL tear (red arrows). Mild joint effusion and peri-ligamentous soft tissue edema are noted. PCL, posterior cruciate ligament; PD-FS, proton density fat-suppressed

A 60-year-old male presented with pain and inability to bear weight after falling while descending stairs. MRI examination revealed a complete tear of the PCL (Figure 6).

Case 4

**Figure 7:**
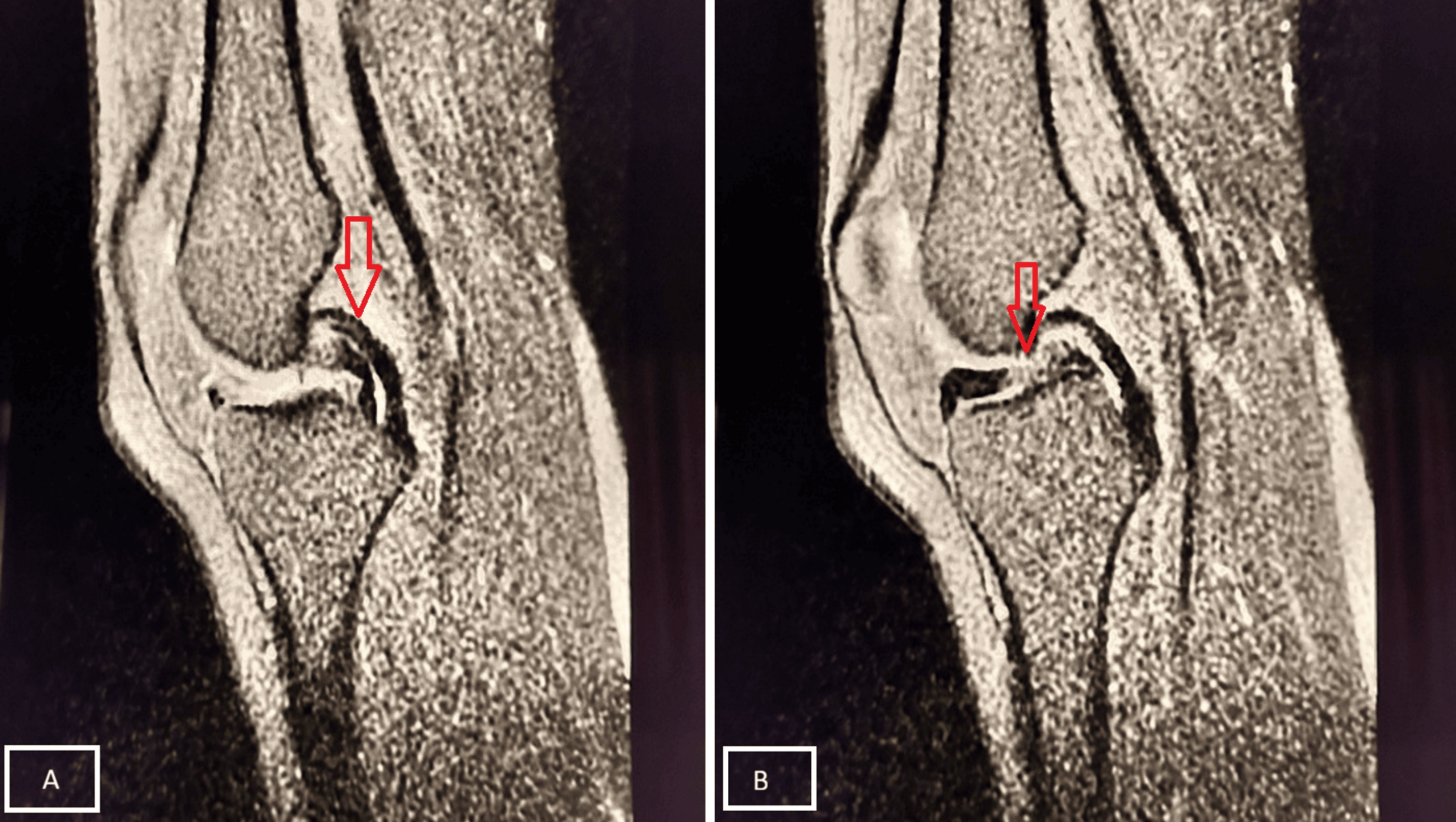
Bucket-handle tear of MM Sagittal PD-FS MRI shows a displaced fragment of the MM anterior to the PCL, producing the “double PCL sign” (A, red arrow). This finding represents a bucket-handle tear (B, red arrow) of the MM (Stoller Grade III meniscal tear). MM, medial meniscus; PD-FS, proton density fat-suppressed

A 21-year-old male sustained a twisting injury while playing football, followed by severe pain and swelling of the right knee. MRI demonstrated an MM tear (Figure 7).

Case 5

**Figure 8:**
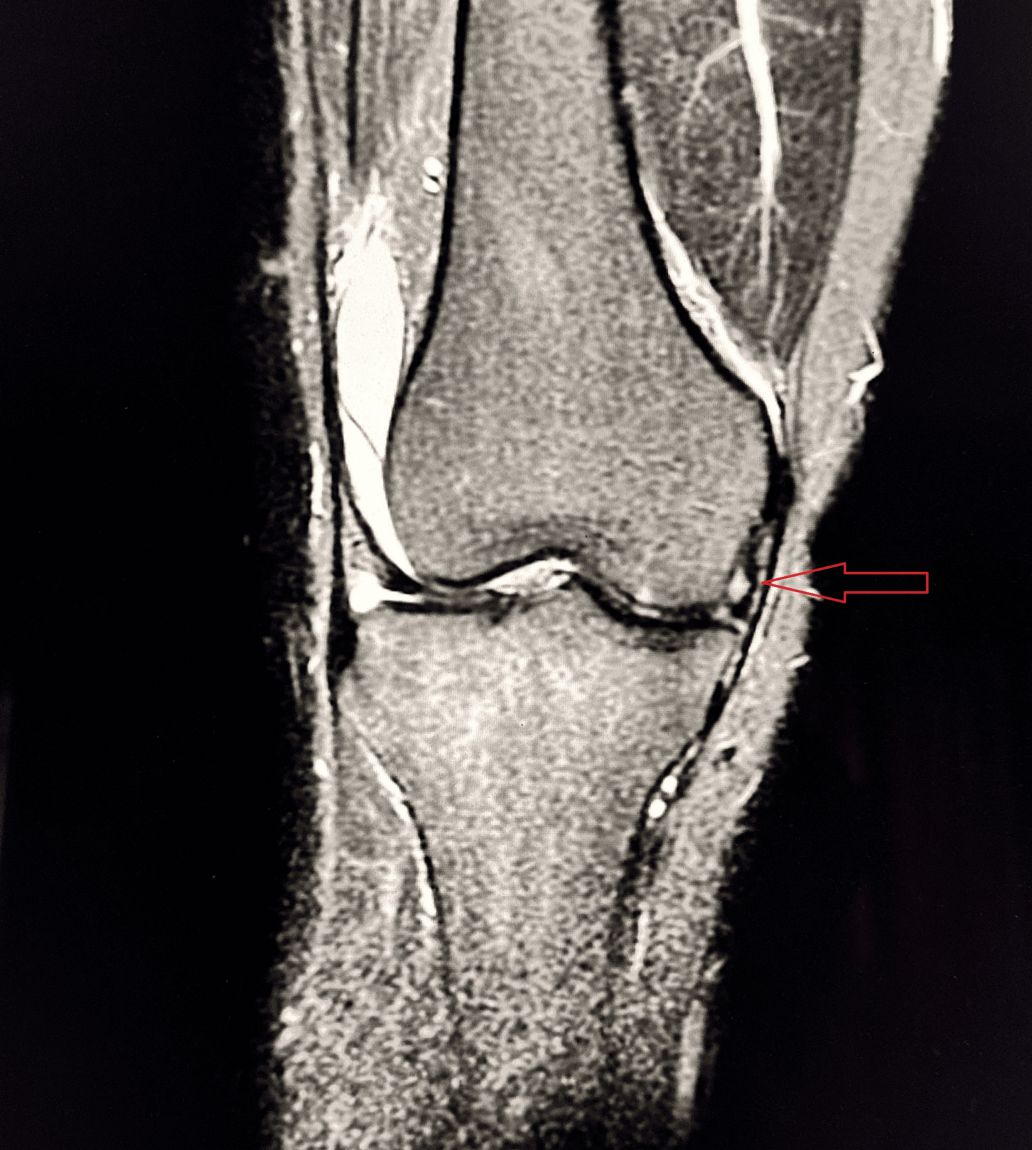
Grade I injury of the MCL Coronal T2-weighted MRI demonstrates thickening and hyperintensity of the MCL without fiber discontinuity, consistent with Grade I MCL sprain (red arrow). Mild joint effusion is present. MCL, medial collateral ligament

A 28-year-old male sustained trauma to the right knee during a riot after being struck by a rod. Clinical examination revealed a positive valgus stress test, suggesting MCL injury. MRI confirmed a Grade I MCL injury (Figure 8).

Case 6

**Figure 9:**
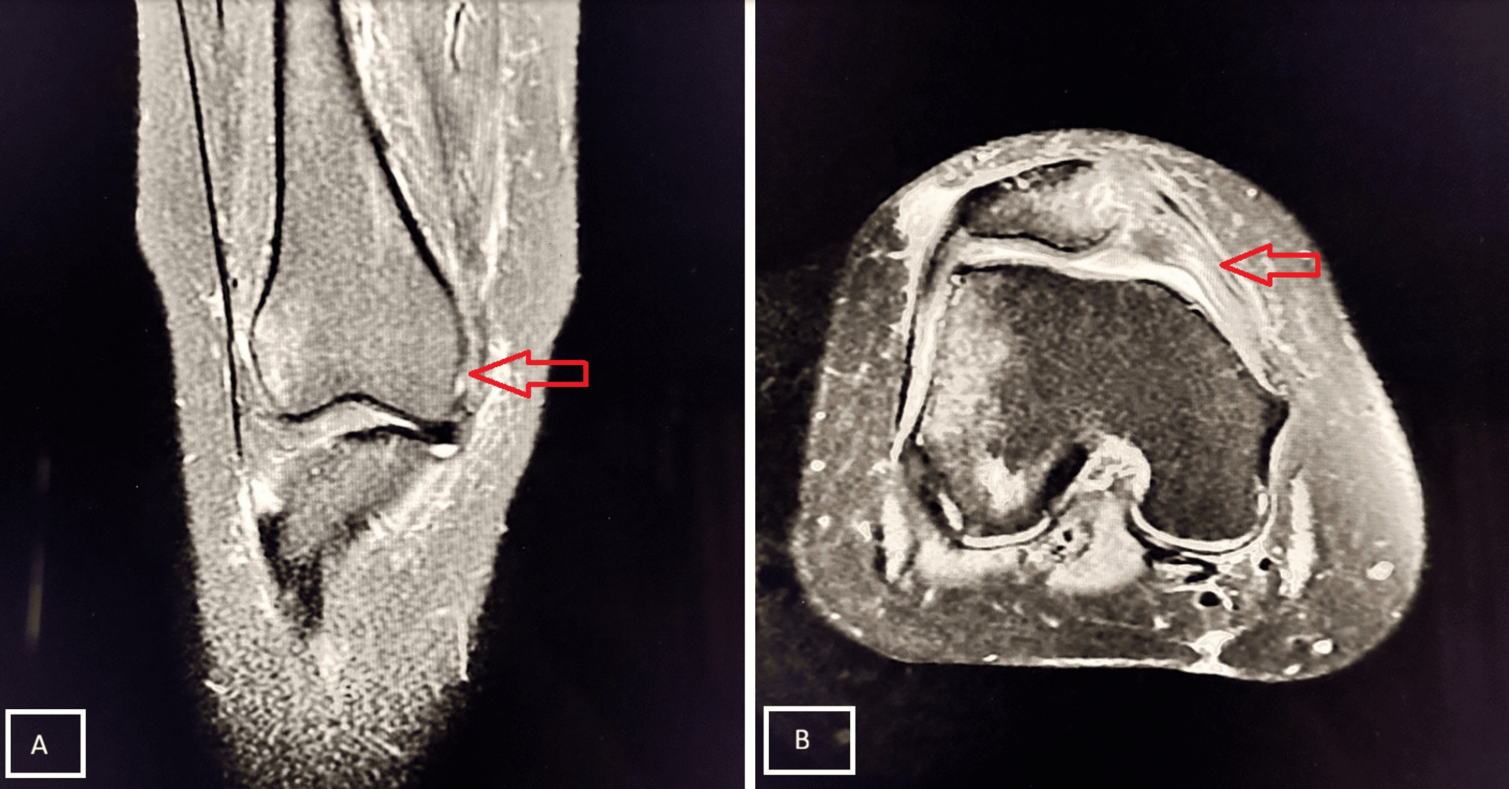
Grade II tear of the medial patellar retinaculum Coronal STIR (A) and axial PD-FS (B) MRI images demonstrate hyperintense signal and partial disruption of fibers of the medial patellar retinaculum, associated with marrow edema of the patella and mild joint effusion, consistent with Grade II injury (red arrows). PD-FS, proton density fat-suppressed; STIR, short tau inversion recovery

A 30-year-old female sustained a direct blow from a hockey ball to the medial aspect of the right knee. Radiographs showed no fracture. MRI revealed a partial Grade II tear of the medial patellar retinaculum (Figure 9).

Case 7

**Figure 10:**
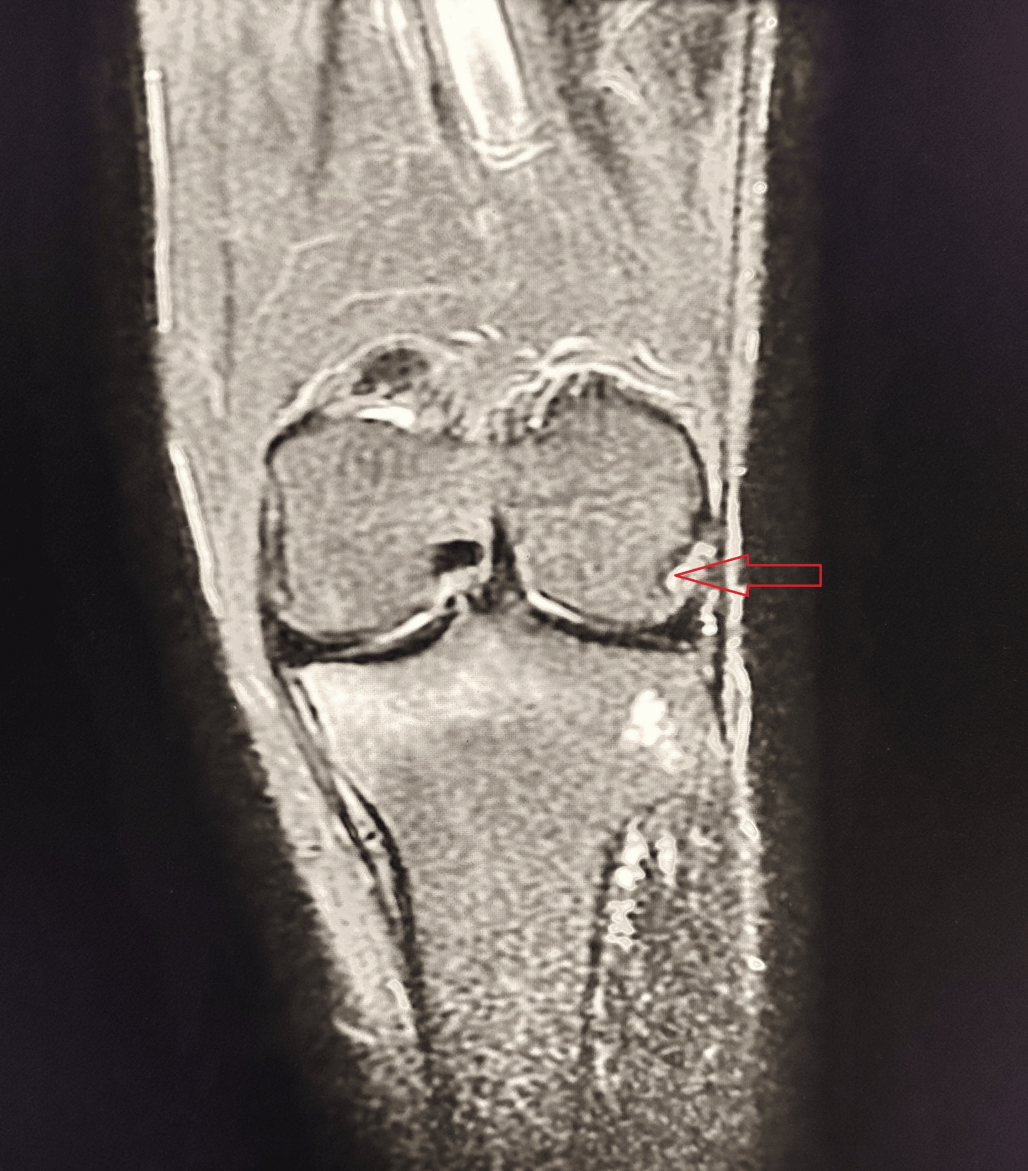
Grade II osteoarthritis of the knee joint Coronal T2-weighted MRI demonstrates reduced joint space in the medial and lateral compartments with marginal osteophyte formation along the femoral condyles and tibial plateau, consistent with KL Grade II osteoarthritis (red arrow). KL, Kellgren-Lawrence

A 52-year-old male presented with morning stiffness, crepitus, and reduced range of motion of the left knee. MRI findings were consistent with degenerative osteoarthritis (Figure 10).

Case 8

**Figure 11:**
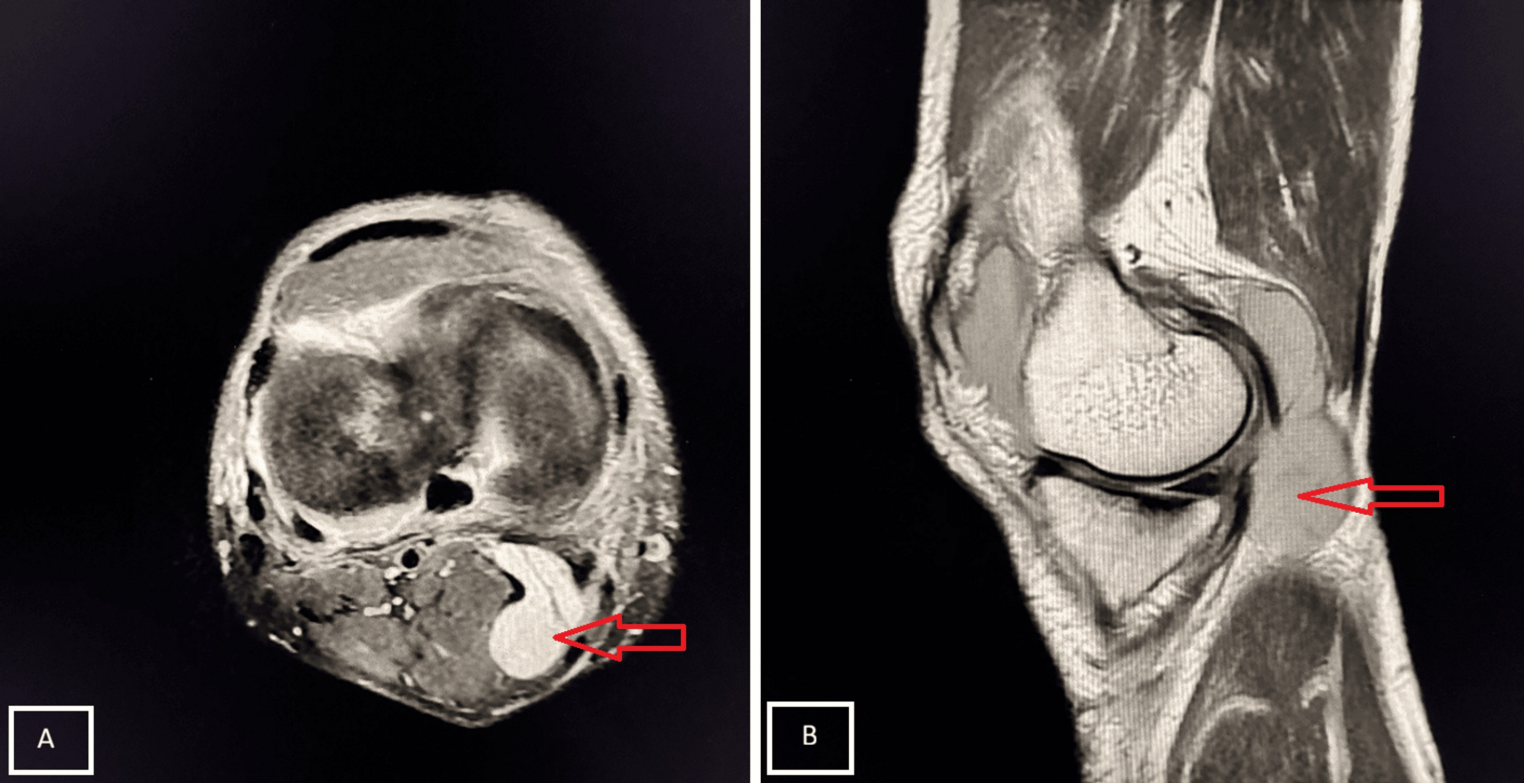
Baker’s cyst in the popliteal fossa Axial PD-FS (A) and sagittal (B) T1-weighted MRI demonstrate a well-defined cystic lesion with internal septations located between the semimembranosus tendon and medial head of the gastrocnemius, communicating with the knee joint via a narrow neck, consistent with Baker’s cyst (red arrows). PD-FS, proton density fat-suppressed

A 58-year-old female presented with pain and swelling in the popliteal region. Clinical examination revealed a palpable mass in the popliteal fossa. MRI confirmed Baker’s cyst (Figure 11).

Case 9

**Figure 12:**
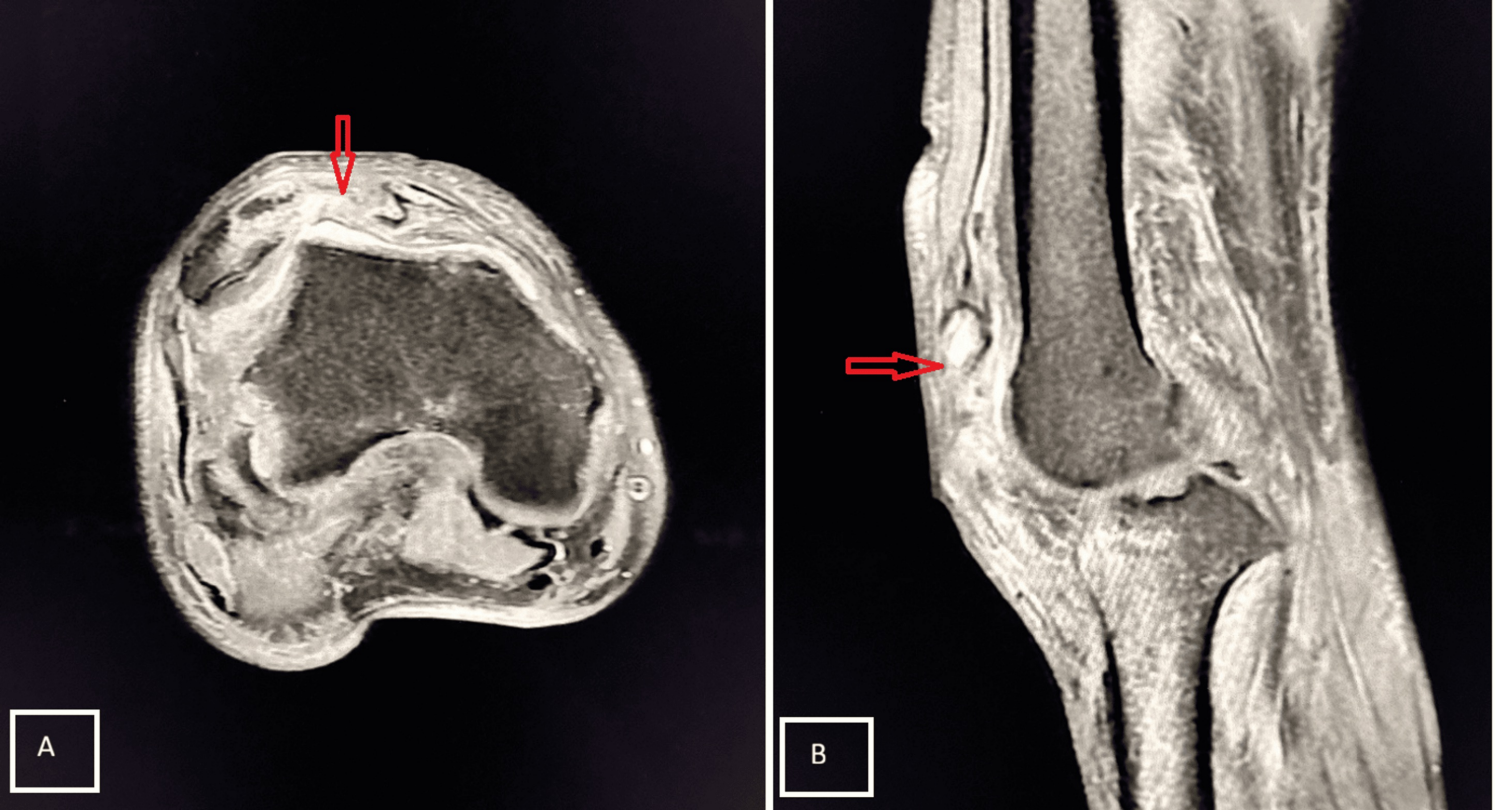
Transverse fracture of the patella Axial (A) and sagittal (B) PD-FS MRI demonstrate a transverse fracture of the patella with marrow edema, associated with mild joint effusion and surrounding soft tissue edema (red arrow). PD-FS, proton density fat-suppressed

A 48-year-old male presented after falling from a two-wheeler, with severe knee pain and inability to extend the right lower limb. MRI revealed a patellar fracture (Figure 12).

Case 10

**Figure 13:**
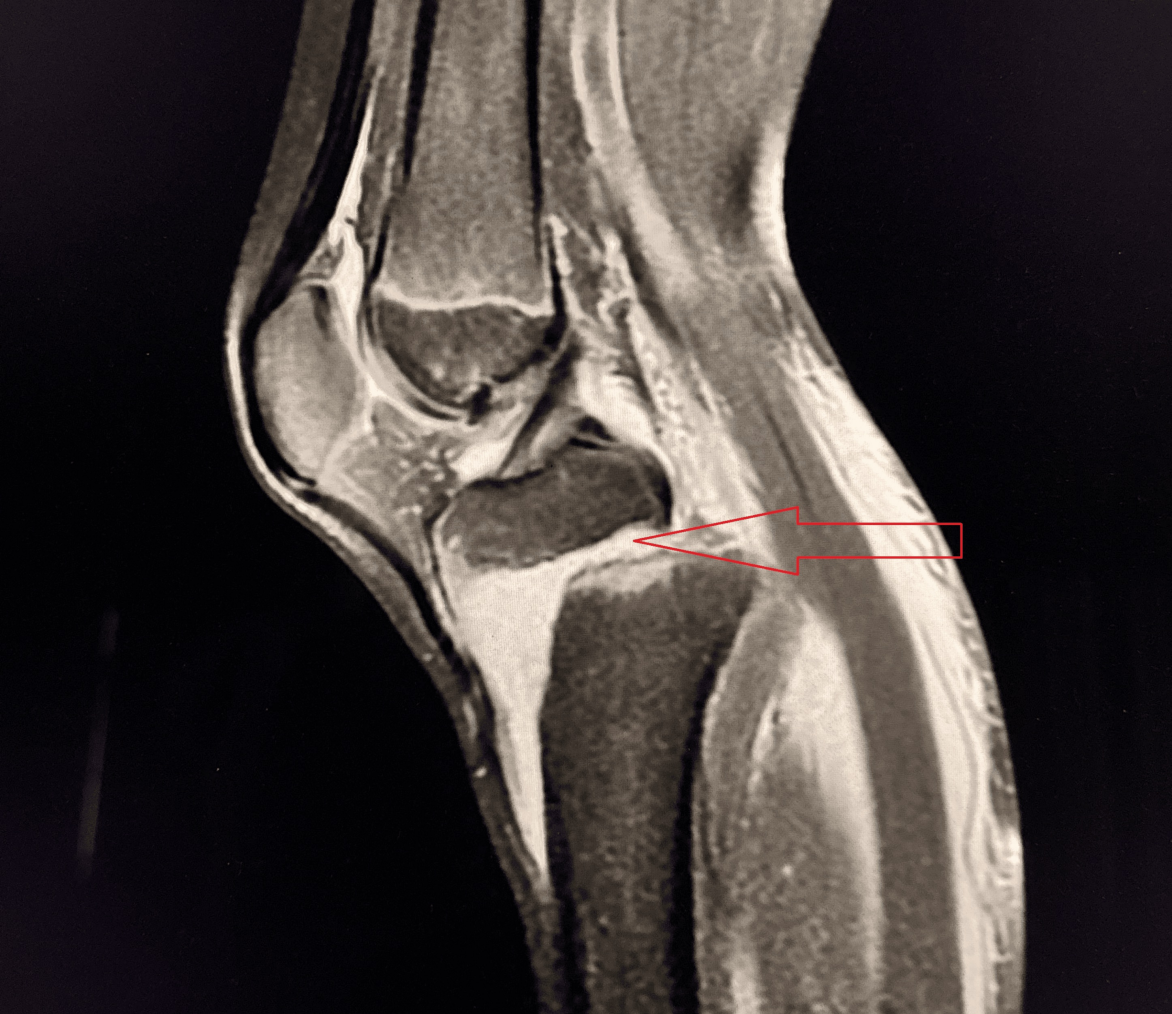
Salter-Harris Type III physeal fracture Sagittal PD-FS MRI demonstrates a fracture line (red arrow) extending through the epiphysis and growth plate, with anterior displacement of the distal fragment and moderate joint effusion. PD-FS, proton density fat-suppressed

A 16-year-old boy presented after a fall from height with severe knee pain and deformity. MRI demonstrated a Salter-Harris Type III epiphyseal fracture of the tibia (Figure 13).

Case 11

**Figure 14:**
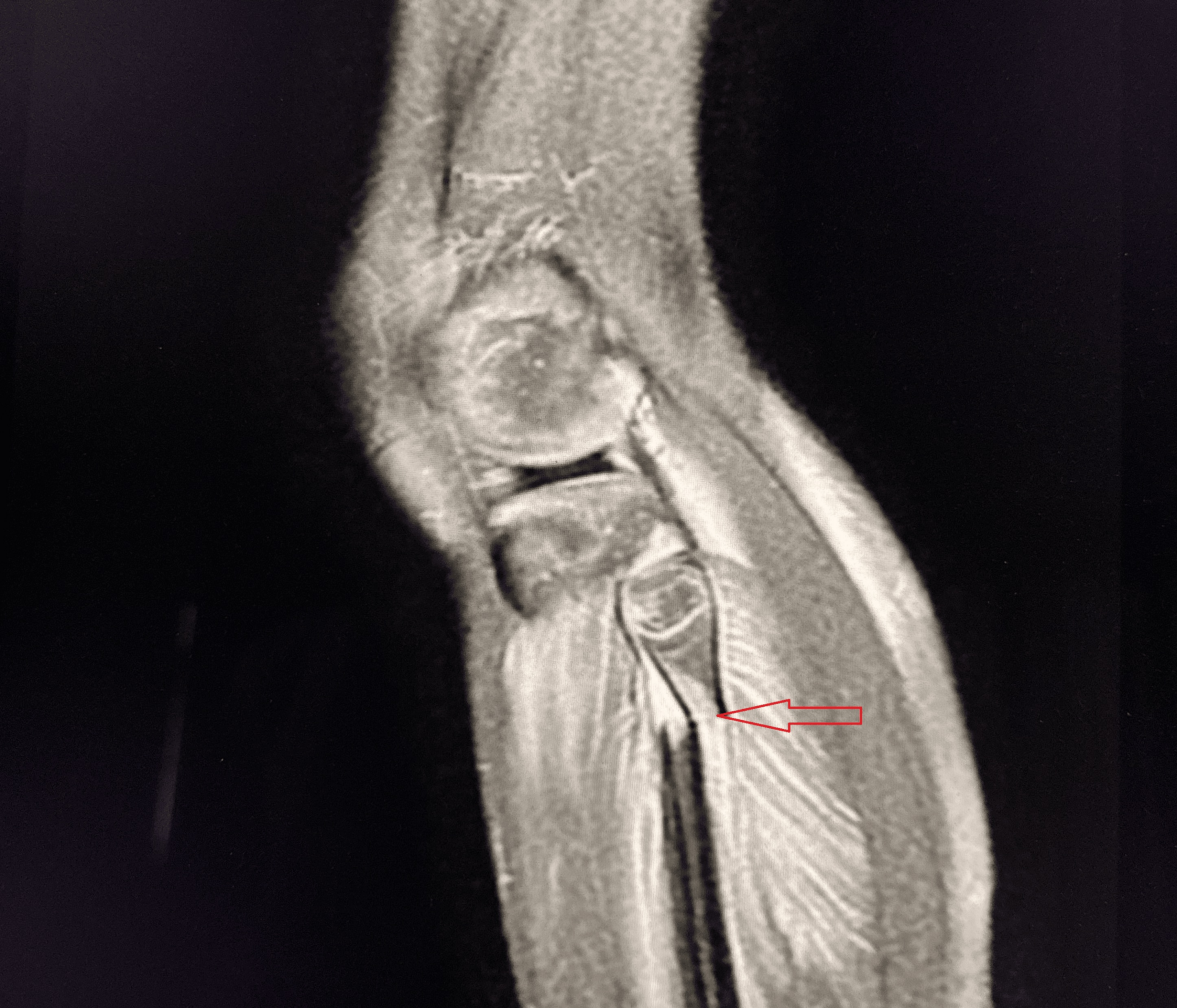
Transverse fracture of fibular neck Sagittal PD-FS MRI demonstrates a transverse fracture of the fibular neck (red arrow) with posterior displacement of the proximal fragment, associated with marrow edema and surrounding muscular edema. PD-FS, proton density fat-suppressed

A 17-year-old male sustained injury to the lateral knee during a bicycle accident, followed by the development of foot drop, indicating possible common peroneal nerve injury. MRI revealed a fibular neck fracture (Figure 14).

Case 12

**Figure 15:**
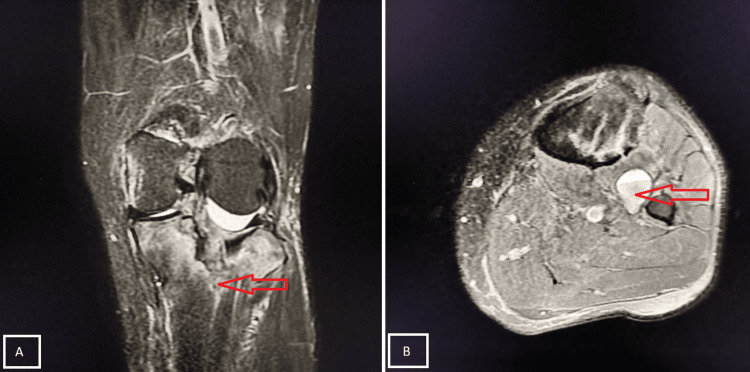
Schatzker Type II tibial plateau fracture Coronal STIR (A) MRI demonstrates a depressed fracture of the lateral tibial plateau (red arrow) with marrow edema and mild joint effusion consistent with Schatzker Type II fracture. Axial PD-FS (B) MRI shows the presence of an associated aneurysmal cyst (red arrow). PD-FS, proton density fat-suppressed; STIR, short tau inversion recovery

A 45-year-old male presented with trauma to the knee after jumping across a small stream. MRI demonstrated a depressed fracture of the tibial plateau (Figure 15).

Case 13

**Figure 16:**
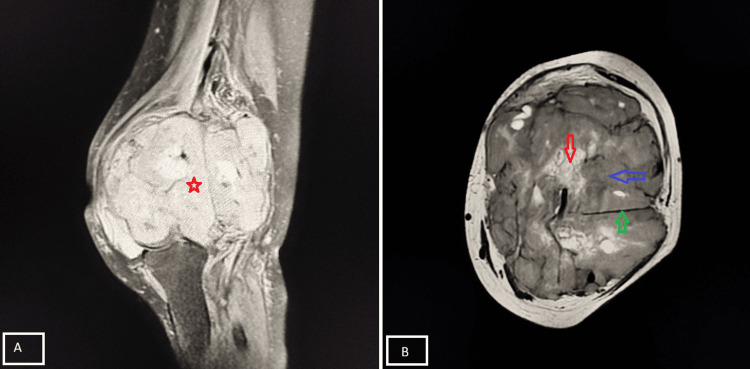
Synovial sarcoma demonstrating the “triple sign” Sagittal PD-FS MRI (A) demonstrates the presence of a heterogeneous multilobulated soft tissue mass (red star). In axial T2-weighted MRI (B), there is the presence of high (red arrow), intermediate (blue arrow), and low (green arrow) signal intensities, representing fluid/necrosis, solid tumor tissue, and fibrous septa, respectively, producing the characteristic “triple sign” of synovial sarcoma. PD-FS, proton density fat-suppressed

A 26-year-old female presented with painless swelling around the knee joint for one year, with gradually progressive restriction of movement. MRI findings were suggestive of synovial sarcoma (Figure 16).

Case 14

**Figure 17:**
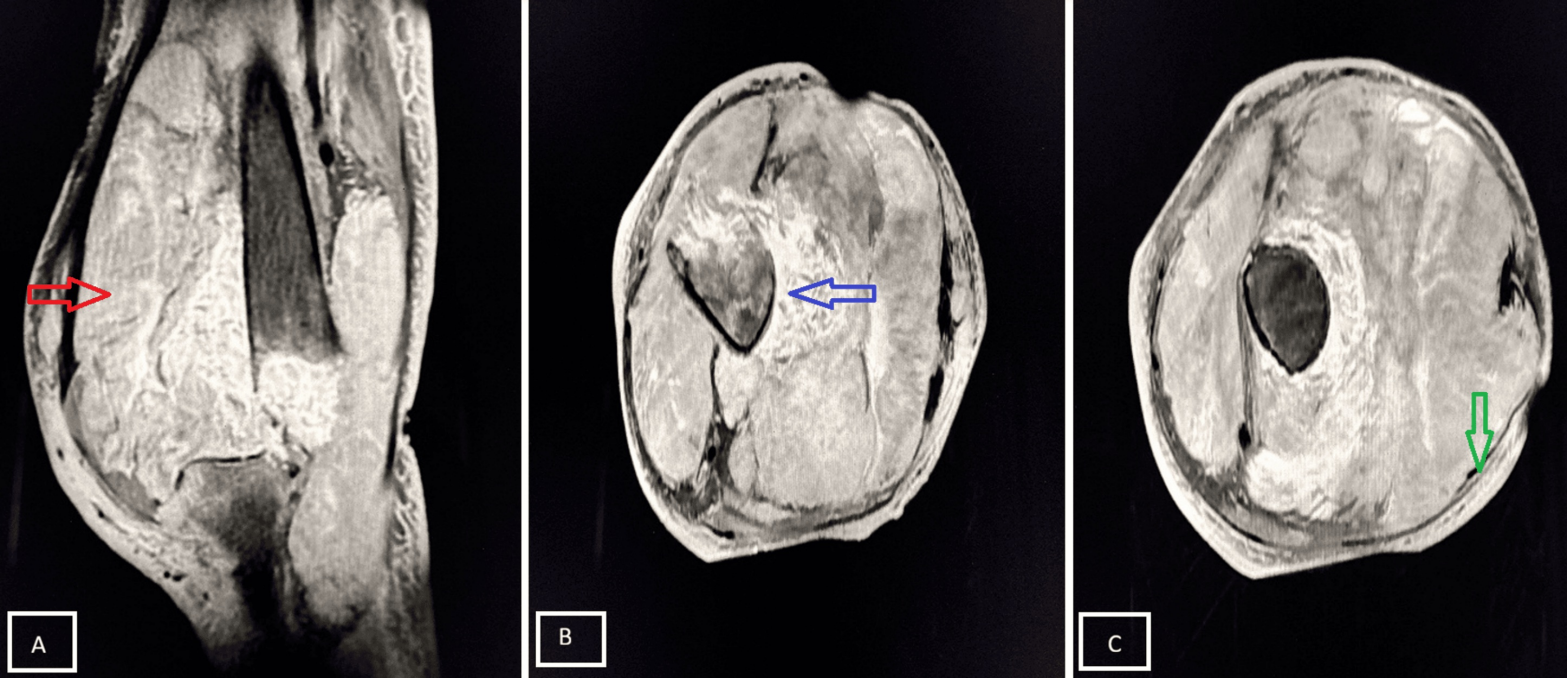
Malignant pleomorphic sarcoma around the knee joint Sagittal PD-FS MRI (A) demonstrates a heterogeneous soft tissue mass arising from the distal femur (red arrow) with cortical destruction and reactive bone formation, with extension into surrounding soft tissues. Axial PD-FS MRI (B) shows areas of high signal intensity (blue arrow), which represent myxoid components, while in the same imaging, a peripheral hypointense rim (green arrow) suggests the presence of a pseudo capsule. Findings are consistent with pleomorphic sarcoma. PD-FS, proton density fat-suppressed

A 60-year-old male presented with progressive swelling around the left knee for nine months, with restricted joint movement. MRI demonstrated a malignant pleomorphic sarcoma arising from the distal femur (Figure 17).

## Discussion

The present study provides a comprehensive evaluation of the spectrum of knee joint pathologies identified on MRI in a tertiary care setting. A male predominance was observed, with the highest incidence among males in the third and fourth decades, whereas female patients were more commonly affected in the fifth and sixth decades. This pattern is consistent with previously reported epidemiological trends, where traumatic knee injuries are more frequent among younger, physically active males, while degenerative conditions are more prevalent in older females [19]. Minor variations in age and sex distribution compared to other studies may reflect differences in population characteristics and referral patterns.

A substantial proportion of patients in this study were overweight or obese, highlighting the potential contribution of increased body mass to knee joint pathology. While a higher prevalence of degenerative conditions was observed in individuals with elevated BMI, particularly among older females, this finding should be interpreted as a descriptive trend rather than a statistically established association. Similar observations have been reported in prior studies, which describe obesity as a contributing factor in knee joint disorders, particularly osteoarthritis [1,4].

Among the various pathologies evaluated, ligamentous and meniscal injuries constituted the most common category, followed by osteoarthritis, cystic lesions, fractures, and neoplastic conditions. This distribution is comparable to that reported by Elzaki et al., although differences in specific lesion categories may reflect variations in study design and inclusion criteria [20]. The presenting clinical features, including pain, swelling, restricted movement, and crepitus, were also consistent with previous literature [2,4].

Ligament injuries and meniscal tears were the most frequent abnormalities in this cohort. ACL tears were the most common, followed by MM tears, which aligns with the findings of Maheshwari et al. [19]. However, other studies have reported MM tears as the predominant lesion, highlighting variability in injury patterns across populations [21]. A higher frequency of ACL, MM, and MCL injuries was observed among younger individuals, whereas PCL and LM injuries were relatively more common in older patients. These observations likely reflect differences in injury mechanisms and degenerative processes, although causal relationships cannot be inferred from this study design. Similar variations have been described in previous studies [20,22].

With respect to injury severity, most MCL injuries were graded as Grade I, ACL injuries predominantly as Grade II, while PCL and meniscal tears were more frequently classified as Grade III. These findings differ from those reported by Shetty et al., who observed a lower proportion of complete meniscal tears, indicating possible differences in patient selection or injury characteristics [23]. Medial retinacular injuries were identified in a small number of cases and were associated with trauma. Unlike some reports where such injuries are commonly linked to patellar dislocation or fractures, no consistent association was observed in this study [24].

Osteoarthritis was the second most common pathology identified. It was more frequently observed in older individuals and showed a higher prevalence among females. While a higher proportion of osteoarthritis cases was noted among overweight and obese individuals, this did not demonstrate statistical significance and therefore should not be interpreted as evidence of a direct association. The observed demographic pattern is consistent with previous studies describing the influence of age, sex, and mechanical factors on degenerative joint disease [25].

A history of prior knee trauma or ligament injury was noted in several cases of osteoarthritis; however, this observation should be interpreted cautiously. Given the retrospective and cross-sectional design of the study, no temporal or causal relationship between prior injury and subsequent osteoarthritis can be established. This finding is therefore presented as an observational association rather than evidence of secondary osteoarthritis [26].

Cystic lesions, including Baker’s cysts, were identified in a subset of patients, most commonly in the fifth and sixth decades. Although a number of these patients had a history of ligament injury or underlying joint pathology, this should be interpreted as a descriptive observation rather than a statistically validated association. Similar findings have been reported in previous studies [5,20].

Fractures around the knee joint were less frequent, with patellar fractures being the most common pattern in this cohort. This differs from studies such as that by Singhi et al., where tibial plateau fractures were more prevalent, possibly reflecting differences in mechanisms of injury and patient populations [27]. Other fracture types, including tibial epiphyseal, tibial plateau, and fibular neck fractures, were identified in smaller numbers, consistent with previously reported distributions [28].

Neoplastic lesions were the least common category. Synovial sarcoma was the most frequently observed malignant lesion, followed by pleomorphic sarcoma and other soft tissue sarcomas. MRI was useful in delineating lesion characteristics such as anatomical location, margins, internal composition, and extent of local involvement. However, given the small number of cases and the absence of complete histopathological and molecular correlation, no definitive conclusions can be drawn regarding tumor characteristics or underlying biological mechanisms. Previous studies have also reported variability in the distribution of malignant knee lesions [20,29]. Importantly, speculative interpretations regarding molecular or chromosomal abnormalities have been avoided, as they are not supported by the available data.

While MRI enabled detailed evaluation of a wide range of knee joint pathologies in this study, it is important to emphasize that the present study does not assess diagnostic accuracy or compare MRI findings with arthroscopy or histopathology. The findings of this study should be interpreted in light of its limitations. The single-center design and retrospective nature may limit generalizability and preclude longitudinal assessment of disease progression or outcomes. In addition, the absence of interobserver reliability analysis, advanced cartilage imaging, synovial assessment, and systematic evaluation of bone marrow edema represent methodological constraints. The exclusion of inpatient cases may also have resulted in underrepresentation of high-energy trauma. Furthermore, the small number of neoplastic cases limits meaningful statistical analysis of this subgroup.

Despite these limitations, the study provides valuable descriptive data on the distribution and imaging characteristics of knee joint pathologies in a tertiary care setting. These findings contribute to the existing literature by offering population-specific insights and highlighting the broad spectrum of pathologies that can be evaluated using MRI in routine clinical practice. The results should be interpreted as observational and descriptive, complementing existing evidence rather than establishing new diagnostic or causal conclusions.

## Conclusions

Ligamentous and meniscal injuries were the most frequent knee joint pathologies, with a statistically significant predominance among younger male patients, and ACL tears representing the most common individual injury. Degenerative pathology, particularly osteoarthritis, was more frequently observed in older individuals with a significant female predominance, while a higher prevalence among overweight and obese patients was noted as a descriptive trend without statistical significance. Fractures were less common, with patellar fractures constituting the predominant pattern, whereas neoplastic lesions were rare, with synovial sarcoma being the most frequently identified malignant entity. Distinct grading patterns across ligamentous and meniscal injuries highlight variability in injury severity. Within the limitations of this retrospective observational design, MRI enabled comprehensive characterization of diverse knee joint pathologies; however, no conclusions regarding diagnostic accuracy or clinical impact can be drawn in the absence of comparative or outcome-based data. Overall, these findings provide population-specific insights into the distribution and imaging patterns of knee joint pathologies and may serve as a basis for future prospective and comparative research.
